# Spontaneous pneumothorax as unusual presenting symptom of COVID-19 pneumonia: surgical management and pathological findings

**DOI:** 10.1186/s13019-020-01343-4

**Published:** 2020-10-12

**Authors:** Roberto Bellini, Maria Chiara Salandini, Serena Cuttin, Stefania Mauro, Paolo Scarpazza, Christian Cotsoglou

**Affiliations:** 1 Department of Surgery, General Surgery Division, Vimercate Hospital, Vimercate, Italy; 2 Department of Pathology, Vimercate Hospital, Vimercate, Italy; 3 Milan Study University, Milan, Italy; 4 Department of Pneumology, Vimercate Hospital, Vimercate, Italy

**Keywords:** Pneumothorax, COVID-19, Thoracoscopy, Case report, Bullectomy, Pathology

## Abstract

**Background:**

Spontaneous pneumothorax has been reported as a possibile complication of novel coronavirus associated pneumonia (COVID-19). We report two cases of COVID-19 patients who developed spontaeous and recurrent pneumothorax as a presenting symptom, treated with surgical procedure. An insight on pathological finding is given.

**Case presentation:**

Two patients presented to our hospital with spontaneous pneumothorax associated with Sars-Cov2 infection onset. After initial conservative treatment with chest drain, both patients had a recurrence of pneumothorax during COVI-19 disease, contralateral (patient 1) or ipsilateral (patient 2) and therefore underwent lung surgery with thoracoscopy and bullectomy. Intraoperative findings of COVID-19 pneumonia were parenchymal atelectasis and vascular congestion. Lung tissue was very frail and prone to bleeding.

Histological examination showed interstitial infiltration of lymphocytes and plasma cells, as seen in non specific interstitial pneumonia, together with myo-intimal thicknening of vessels with blood extravasation and microthrombi.

**Conclusions:**

Although rarely, COVID-19 may present with spontaneous pneumothorax. Lung surgery for pneumothorax in COVID-19 patients can be safely and effectively performed when necessary.

## Introduction

On December 2019, a novel coronavirus-related pneumonia has been first reported in the city of Wuhan, China [1]. Since then, the disease has spread worldwide throughout Asia, Europe, America and Africa. In Italy, the first cases of novel coronavirus infections were reported on February 21, 2020.

Patients with COVID-19 can have mild symptoms such as fever, cough, fatigue and smell and taste dysfunction, or more serious and rapidly progressing respiratory impairment such as Acute Respiratory Distress Syndrome [2, 3].

Radiological presentation at CT scan is characterized by bilateral interstitial infiltrates with ground glass opacities (GGO), multiple lobar and subsegmental consolidations and air bronchograms [4]. Other findings such as pleural effusion, cavitation, pneumomediastinum and pneumothorax are uncommon and in some cases anecdotal [4–6].

Pneumothorax is often a late manifestation of the disease, mainly due to patient intubation and positive air-pressure oxygen administration [7].

We describe two cases of spontaneous pneumothorax (SP) as presenting symptom of COVID-19 disease and their surgical management, with insight on intraoperative and pathological findings.

### Case 1

**Fig. 1:**
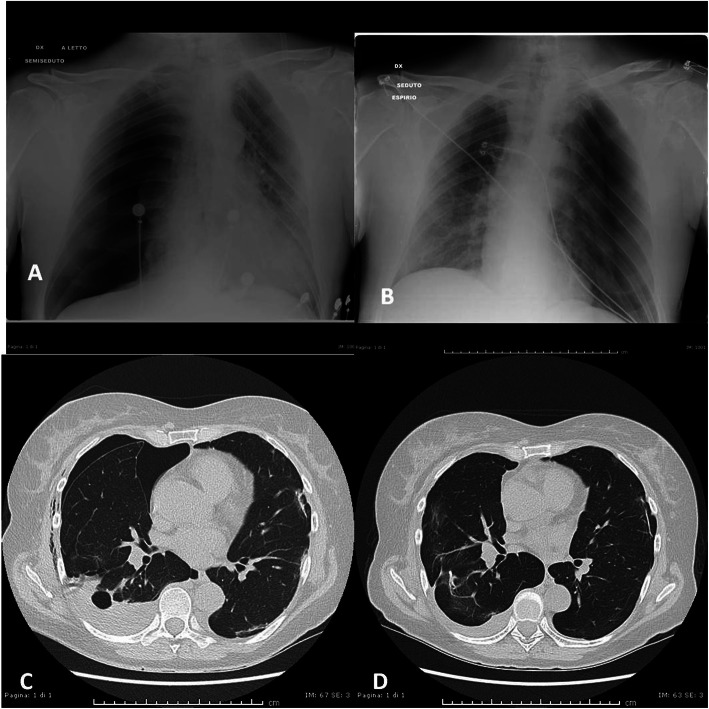
Chest X-ray showing massive left (**a**) and right (**b**) pneumothorax in COVID-19 patient 1; preoperative CT scan showing right pneumothorax with bulla of the apical segment of the lower lobe (**c**) and ground glass opacities (**d**) typical of COVID-19 disease in patient 2

A 58-year-old male presented to the Emergency Department of our hospital on March 22 for sudden right thoracic pain, dyspnea, and mild fever. He was a former smoker, with no significant medical history. At presentation, he had tachycardia and hypertension (heart rate 120, blood systemic pressure 190/120), tachypnea and desaturation (respiratory rate 40/min, arterial blood saturation 81%). At hemogasanalysis, pH was 7.38, paO2 was 53 mmHg and paCO2 44 mmHg. Chest X-ray showed massive right pneumothorax (Fig. 1a): a 20 Ch chest drain was positioned, with immediate relief of patient dyspnea and normalization of vital parameters. Post-procedural X-ray showed full re-expansion of the lung. Blood tests revealed mild leukocytosis with lymphopenia, slight lactate dehydrogenase (LDH) elevation, normal C-reactive protein (CRP). A CT scan showed bilateral interstitial pneumonia with GGO and air bronchograms, suggesting COVID-19 disease. Oropharyngeal swab test was positive for Sars-CoV2.

The patient was treated with broad spectrum antibiotics (ceftriaxone and azythromycin), hydroxychloroquine, intra venous fluids, and nasal high-flow rate oxygen. Chest drain was removed on day 5, after seeing gradual regression of pulmonary infiltrates and no recurrence of pneumothorax at follow-up X-ray. The patient was discharged 3 days later.

Twenty-eight days later the patient was readmitted to our hospital for sudden dyspnea and chest pain. Chest X-ray showed left (contralateral) massive pneumothorax. A 20 CH chest tube was inserted, with subsequent pulmonary re-expansion (Fig 1b). A second oropharyngeal test was positive for COVID-19. Because of the bilateral occurrence of massive pneumothorax, he was a candidate for surgery. On April 24 the patient underwent left thoracoscopy with multiple pulmonary resections of dystrophic areas and mechanical pleurodesis by surgical pleural scarification and partial pleurectomy along costal arches, from the 3rd to the 7th rib. A single chest drain was left and then removed on postoperative day (POD) 6. The patient was discharged the day after, when routine control chest X-ray showed no recurrence of pneumothorax.

### Case 2

A 53-year-old female presented to the Emergency Department with a three-day history of cough, fever, right chest pain and hemoptysis. The patient had a medical history of blood hypertension, and she was a non smoker. Chest X-ray showed right 35 mm SP, that extended from the apex to the basal edge of the lung, together with bilateral pulmonary infiltrates, suggestive of COVID-19 disease. A 20 Ch left chest drain was inserted in 5th intercostal space, and post-procedural X-ray showed complete re-expansion of the lung. After positioning, no persistent air loss was seen from the drain. Blood tests revealed normal white blood cell count with slight elevation of LDH and CRP. Hemogasanalysis revealed moderate hypoxhemia, with a paO2 level of 67 mmHg, with low carbon dioxide levels (paCO2 25 mmHg) and mild alkalosis (pH 7.48). Oropharyngeal swab test was positive for Sars-CoV2. Chest CT scan showed basal bilateral confluent GGOs with small consolidative opacities, and an 18 mm bulla located at the apical segment of the right lower lobe, within a GGO area (Fig. 1c and d).

The patient was treated with oral hydroxychloroquine, antibiotic therapy with piperacillin-tazobactam, and high flow nasal oxygen.

Chest drain was kept in place with no air loss for a few days, and then removed on day 5. Unexpectedly, routine control chest X-ray before discharge revealed a 35 mm recurrence of right pneumothorax.

Because of the recurrence of pneumothorax, and the consequent risk of adding further respiratory distress in a COVID-19 pneumonia, the patient was a candidate for surgery. Surgical procedure was performed with right dual-port thoracoscopy and consisted in double pulmonary resections (i.e. apicectomy and resection of apical segment of lower lobe) with mechanical pleurodesis by surgical pleural scarification and partial pleurectomy from the 3rd to the 7th costal arch. A single chest drain was left and then removed on POD 6. After removal, control X-ray showed no recurrence of pneumothorax. During the following days, the patient experienced recurrence of fever with mild elevation of white blood cell count, although both chest X-ray and hemogasanalysis did not show deterioration of pneumonia. Therefore, the patient was treated with symptomathic therapy, until spontaneous regression of the fever and normalization of white blood cell count occurred. She was discharged on POD 12.

## Discussion

Pneumothorax, usually due to prolonged ventilation with positive pressure, is a relatively common complication of COVID-19 pneumonia, affecting up to 5.9% of patients [7].

In 2004, Sihoe et al. reported a 1.7% incidence of SP in Severe Acute Respiratory Syndrome (SARS) patients [8]. In this series, pneumothorax was a late complication of SARS, occurring from 14 to 37 days after initial diagnosis, suggesting that a sustained period of lung inflammation is first required. Four patients were treated with chest drain, and two were managed conservatively. Chest drains were removed from 14 up to 31 days after insertion: permanence of drains was mainly due to prolonged air loss. None of the patients underwent surgical operation because of both severe impairment of patient’s lung function and high anesthetic risk, and because of concern for infection risk to operating room staff [8].

As for novel coronavirus disease, other authors have already reported SP as a clinical manifestation for COVID-19 [4, 5, 9, 10]; however, none of them was managed with a surgical approach.

Tian and colleagues [11] reported of two patients submitted to pulmonary lobectomy for lung cancer who developed fever and respiratory failure after surgery: a postoperative oropharyngeal swab test revealed a Sars-CoV2 infection. A review of preoperative CT scan revealed bilateral GGOs in one of them, while the second developed radiologically detectable interstitial pneumonia on POD 2. This is actually the first report of COVID-19 patients with lung pneumonia submitted to lung surgery.

We report the first two cases of patients with novel coronavirus pneumonia with SP as presenting symptoms who were surgically treated.

Our first approach for both patients was chest drain, because we had no knowledge of whether COVID-19 could favor complications after surgery. However, after recurrence of SP in both patients we had to consider surgical treatment because of the need to avoid possible further respiratory distress in patients with ongoing interstitial pneumonia.

**Fig. 2:**
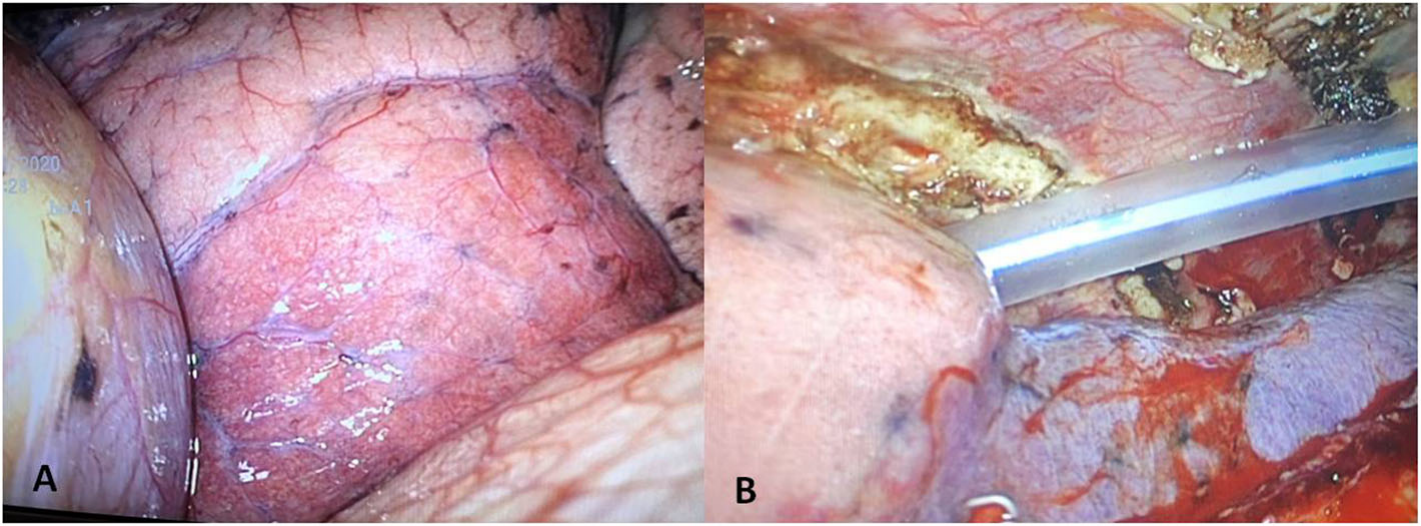
Intraoperative finding of parenchymal vascular congestion and atelectasis in patient 1 (**a**) and in patient 2 in the site of bullectomy at the apical segment of lower lobe (**b**)

Intraoperative findings were of segmental and subsegmental areas characterized by atelectasis and vascular congestion, which proved to be very frail at manipulation and traction. These areas corresponded to GGO infiltrates at preoperative CT scan (Figs. 1 and 2). In particular, the rupture of the bulla at the apical segment of right lower lobe within an area of important vascular congestion and pulmonary atelectasis was probably the cause of patient 2 hemoptysis before hospital admission (Fig. 1c).

Notably, despite regular coagulation parameters and no anticoagulant therapy, both patient had a tendency for bleeding during gentle manipulation, so that we preferred not to perform extensive pleural scarification. This finding may be explained by systemic inflammation, which can impair proper coagulation [12].

Finally, during pulmonary re-ventilation at the end of the procedure, the COVID-19 atelectasis areas did not re-expand as expected, due to loss of parenchymal compliance, despite the use of positive pressure air flows.

At histological specimen evaluation the main finding was an interstitial pneumonia with ongoing reparative processes, associated with vascular changes probably responsible for increased vascular resistance. In particular, intense, diffuse and uniform chronic inflammation was located in the septal interstitium, with prevalence of lymphocytes and plasma cells, as typically found in non specific interstitial pneumonia. Extensive endoalveolar fibroblastic overhangs located in the centrolobular seat were retrieved. Alveolar spaces were filled with rare alveolar macrophages and proteinaceous exudates. Signs of viral infection consisted of nuclear pseudoinclusions, indicating cytopathic effects.

**Fig. 3:**
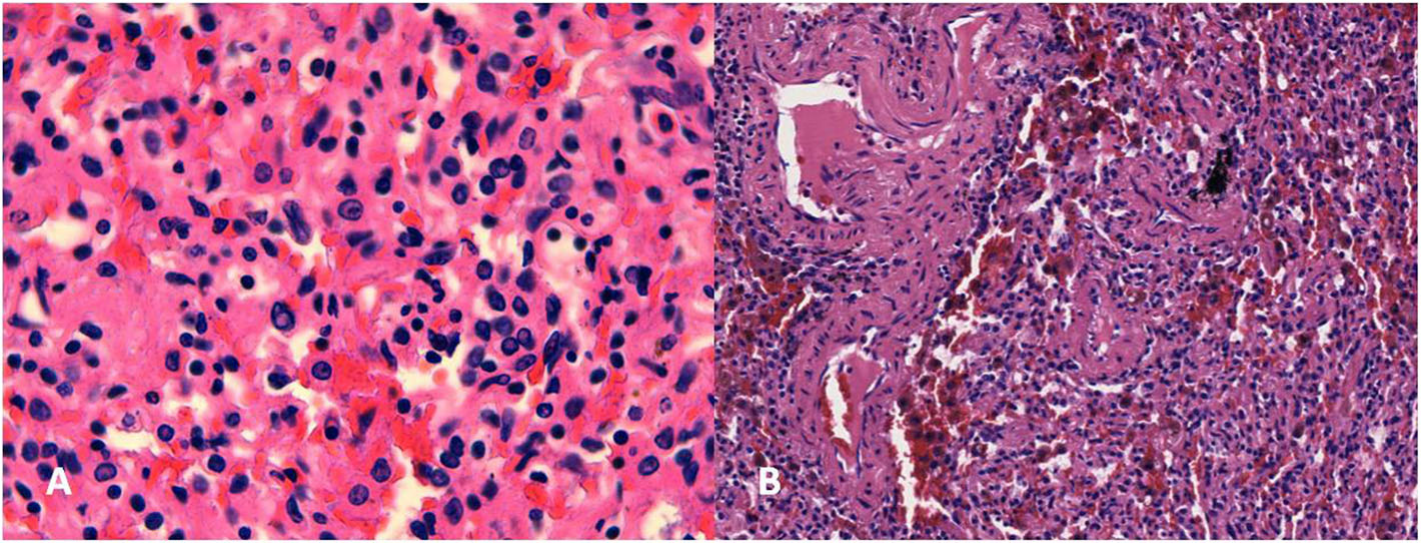
Histological specimen. EE 400x viral cytopathic effects: nuclear pseudoinclusions (**a**). EE 200x vascular microthrombi associated with reduction of vascular lumen (**b**)

Vascular changes were of multiple and extensive interstitial and endoalveolar blood extravasations, and marked myo-intimal thickening with associated blood stasis, sometimes with microthrombi. Subendothelial infiltration of lymphocytes, suggestive of endotelitis, was also retrieved (Fig. 3).

Lastly, areas of distal emphysema and subpleural bullous formations with hemorrhagic content were also observed.

These findings are comparable to what Tian has described [11].

Although other authors have described SP as a clinical manifestation of SARS-Cov2 infection, this is the first report of diagnosed COVID-19 patients who underwent surgical treatment for spontaneous pneumothorax: intraoperative findings of rotten bulla in a COVID area explained why conservative treatment had failed in patient 2, as well as similar findings of dystrophic and fragile parenchyma within COVID areas for patient 1.

Differently from SARS patients [8], in these cases pneumothorax was the first symptom of COVID-19 and occurred at the beginning of pneumonia. Moreover, none of them experienced severe respiratory impairment requiring invasive ventilation. This fact may reflect a different mechanism of pneumonia, where lung rupture does not occur as a final stage of prolonged inflammation, but is an immediate consequence of virus infection in selected patients: microvessel inflammation and capillary wall thickening at the early stages of the disease may play a significant role in this process, for which further studies are needed.

Despite understandable concerns for staff safety, all operative room members wore personal protection equipment as recommended by Italy SSI [13] and none of them developed COVID-19 infection. At 1 month follow up, all staff workers were COVID-19 IgG-free.

## Conclusions

Spontaneous pneumothorax is a possible manifestation of COVID-19 disease.

When conservative treatment fails, surgery can be safely and effectively performed in COVID-19 patients with mild respiratory impairment.

## Data Availability

Not applicable.

## Abbreviations

COVID-19: Novel coronavirus associated pneumonia
GGO: Ground glass opacity
SP: Spontaneous pneumothorax
LDH: Lactate dehydrogenase
CRP: C-reactive protein
SARS: Severe acute respiratory syndrome
POD: Postoperative day
